# The role of motor memory in action selection and procedural learning: insights from children with typical and atypical development

**DOI:** 10.3402/snp.v5.28004

**Published:** 2015-07-08

**Authors:** Jessica Tallet, Jean-Michel Albaret, James Rivière

**Affiliations:** 1 Université de Toulouse 3, PRISSMH EA 4561, Toulouse, France; 2 Université de Rouen, Psy-NCA 4700, Rouen, France

**Keywords:** motor control, motor routine, C-not-B task, developmental coordination disorder

## Abstract

Motor memory is the process by which humans can adopt both persistent and flexible motor behaviours. Persistence and flexibility can be assessed through the examination of the cooperation/competition between new and old motor routines in the motor memory repertoire. Two paradigms seem to be particularly relevant to examine this competition/cooperation. First, a manual search task for hidden objects, namely the C-not-B task, which allows examining how a motor routine may influence the selection of action in toddlers. The second paradigm is procedural learning, and more precisely the consolidation stage, which allows assessing how a previously learnt motor routine becomes resistant to subsequent programming or learning of a new – competitive – motor routine. The present article defends the idea that results of both paradigms give precious information to understand the evolution of motor routines in healthy children. Moreover, these findings echo some clinical observations in developmental neuropsychology, particularly in children with Developmental Coordination Disorder. Such studies suggest that the level of equilibrium between persistence and flexibility of motor routines is an index of the maturity of the motor system.

In the traditional cognitive theories of human memory, memory is the process by which a new material is encoded as a symbolic representation, stored, and eventually retrieved when it is required (Anderson, 1995). Memory is experimentally tested with three steps: firstly, participants are required to practice a novel task with a stimulus which serves as a model. Then, a retention interval corresponds to a rest period during which participants do not practice the task. Finally, the stimulus/model is possibly withdrawn, and participants are invited to recall the practiced task from memory. The memorisation process enables the passage from stimulus-triggered actions to endogenously planned actions, guided by the representation stored in memory. In the case of motor memory, motor behaviours are stored in memory as *motor routines*.

Human motor memory is required to plan, anticipate, select, adapt, learn, recall, and also forget motor behaviours, thanks to a dynamical process by which old/pre-existing and new motor routines interact permanently. Learning enables to store motor routines in long-term memory and, in turn, the stored motor routines can be used to plan new motor routines or to select future actions. Thus, the permanent interplay between new motor routines and those stored in long-term memory explains that motor memory can be both *robust* to maintain persistent motor routines over time or in spite of perturbations and *flexible*, that is able to adapt or inhibit old motor routines to create new routines (Tallet, 2012). This interplay can take the form of a *cooperation* when one motor routine facilitates the acquisition or the retrieval of another motor routine, or a *competition* when one motor routine interferes with the acquisition or the retrieval of another motor routine. Old and new motor routines can influence each other by the intervention of two kinds of memory: proactive and retroactive memory (Schmidt and Young, 1987). *Proactive memory* refers to the fact that an old (previously learnt) motor routine can facilitate or impair the acquisition or retrieval of a new motor routine. For example, when a life-long tennis player begins to learn badminton, he/she may have difficulty in learning the correct forehand routine for badminton because an interference could occur with the previously learnt forehand routine for tennis, which is not exactly the same. *Retroactive memory* operates when a newly learnt motor routine influence the retrieval of an old (previously learnt) motor routine. For example, if one changes the location of his/her keys, he/she may forget the long-used location during few days or weeks.

The link between motor memory, action selection, and programming can be illustrated as follows: during motor learning, assessed by a procedural learning task, a new motor routine is built on the basis of motor routines pre-existing in the motor repertoire (proactive competition/cooperation). After learning, the new motor routine can persist in memory to be used after a long-term delay. Thus, the new motor routine is permanently integrated and consolidated in the pre-existing motor repertoire and can be used (1) to avoid possible motor perseverations, that is, to avoid the repetition an old motor routine, which has become prepotent but not required anymore by environmental constraints and (2) to select and programme the most adapted new motor routine on the basis of environmental constraints.

In this article, we will describe studies on the C-not-B task and procedural learning in typical and atypical development to better understand how motor memory is involved in action selection and programming during childhood.

## Motor memory and C-not-B task in toddlers

The motor memory hypothesis has been evoked to explain an intriguing error in early childhood – the C-not-B error. Toddlers have been found to fail on a three-location search task involving invisible displacements of an object, namely the C-not-B task (see Rivière & Lécuyer, 2003). In this task, a child is shown the experimenter's hand that contains a toy. The experimenter's hand then successively disappears under the three cloths (A, B, and C). The examiner silently releases the toy under the second cloth (B). The hidden object makes a bump in the B cloth that covers it. In this task, the child thus has to issue a reaching movement based on a cue indicating the correct location of a hidden object (i.e. the bump under the B cloth) and to ignore irrelevant information (i.e. the motion of the experimenter's hand that disappears under the C cloth). Children aged from 2.5 years fail this task by being strongly biased towards the last cloth that the experimenter's hand passes under, and this has been labelled the C-not-B error. We proposed that a motor routine prevents healthy toddlers from expressing an appropriate behavioural response in the C-not-B task. This motor routine is the motor tendency to search for things in the direction where they, or more exactly their containers, were last seen. Such a prepotent motor response may preempt full consideration of a visual clue indicating the correct location of the hidden object. This behaviour that disappears at the age of 3 years reflects a lack of flexibility of the youngest children.

The hypothesis that the motor memory plays a key role in generating the C-not-B error is supported by Rivière and Lécuyer's study (2008). In this experiment, we provided some evidence to suggest that toddlers’ performance on the C-not-B task can improve dramatically by putting a 200 g weight on their arms. Indeed, this simple manipulation had a significant impact on performance, with 77% correct performance as compared with 44% in the standard condition. We explained these findings by suggesting that the success in the C-not-B task of toddlers with additional arm weights could result from a disruption of automatic hand movement that is triggered by sensory signals, namely salient features of the C-not-B task.

The results of a recent study (Rivière & David, 2013) further strengthened this interpretation. In three experiments, we investigated the nature of the constraints underlying toddlers’ performance in this task. In Experiment 1, children aged 2.5 years were tested in a new version of the C-not-B task to investigate whether reaching with a detour leads to inhibition of direct visuomotor activation. The findings show that toddlers succeed more in the C-not-B task when a transparent barrier obstructs the path of the reaching movement. The results of Experiment 2 indicate that the successful performance of the children with a barrier cannot merely be the consequence of the longer duration of arm movements. Experiment 3 demonstrated that a simple change in testing procedure, which involved only changing the response option (selecting a location with a stick instead of reaching a location), enabled 2.5-year-olds to succeed more in the C-not-B task. In spite of the fact that response options share a similar action feature (i.e. a movement with the hand), the C-not-B task content may have triggered automatic activation mechanisms only for reaching responses. Visibly, difficulty in inhibiting a prepotent reaching movement is a critical element in toddlers’ performance in the C-not-B task. Taken together, the studies about the C-not-B error suggest that it is a response induced by a motor routine.

The motor routine at work in the C-not-B task may arise from recurring embodied experiences. The frequency with which this behavioural routine is executed appears to determine the strength of neural networks that subserve it. Indeed, the strength of this motor routine appears to be acquired through the gradual reinforcing of neural connections in the course of day-to-day experience (cf. Rivière & Lécuyer, 2003). The neural mechanisms that conspire to produce motor routines begin to be explained. Thus, Erlhagen and Schöner (2002) proposed a neural network account of motor programming. In this theoretical framework, movement parameters are represented by activation fields, distributions of activation defined over metric spaces. The fields evolve under the influence of various sources of localised input representing information about upcoming movements. One such source is a memory trace of activation distributions representing the recent motor history.

The studies discussed above help to understand how motor memory is involved in action selection during childhood. Moreover, they echo some clinical observations in developmental neuropsychology, particularly in children with Developmental Coordination Disorder (DCD). DCD is a neurodevelopmental disorder, which manifests as clumsiness, slowness, and inaccuracy of goal-directed movements, and interferes significantly with many activities of daily living. It is a frequent life-long condition (5–6%) and is not due to an intellectual disability, a visual impairment or to a known neurological condition (DSM-5, American Psychiatric Association, 2013). Impairment in action selection in DCD could be viewed as a default to select the task-relevant motor routine due to a strong persistence of pre-existing action in memory.

## Motor memory and procedural learning in typical and atypical development

Procedural learning also brings information about memory persistence and flexibility and its evolution through childhood. According to the model of Doyon and Benali (2005), procedural learning follows five successive stages in adults: fast learning, slow learning, consolidation, automatisation, and retention. The fast learning stage takes place in the beginning of the repetitive practice in which the fastest and largest improvements in performance occur. Slower and lower improvements are observed with further repetitive practice of the motor routine (slow learning; Adams, 1971). After an interruption of practice of 4–6 hours or sleep, a consolidation stage occurs where performance undergoes either a spontaneous offline increase in performance or an increase in resistance to interference from the learning of a new and a competing similar motor routine (Shadmehr & Brashers-Krug, 1997; Robertson, Pascual-Leone, & Miall, 2004). Then, the task is automatised, which means that the new motor routine can be performed with minimal cognitive resources, that is, with the same level of performance in spite of a concurrent double task. Finally, the new motor routine can be performed with the same level of performance after long delays without further practice (retention stage). Hence, once the new motor routine has undergone all these five stages, it is robust enough to persist permanently in long-term memory without alteration.

The consolidation stage seems to be the most relevant to study the evolution of the competition between the newly learnt motor routine and a new one. Recent studies comparing adults and children highlight developmental changes in the memory consolidation of procedural learning. For example, Dorfberger, Adi-Japha, and Karni (2007) found that procedural learning in children (aged 9–12 years) leads to a new motor routine, which is more resistant to interference from a subsequent learning than in adolescents (aged 17 years). Ashtamker and Karni (2013) suggest that memory consolidation of a newly acquired motor routine is faster in children (aged 9–12 years) than adults (within 1 h vs. 4–6 h post-training). Moreover, Wilhelm, Diekelmann, & Born (2008) demonstrate that children are more susceptible to consolidate offline new motor routines during wakefulness than during sleep, whereas opposite results are found in adults. All these results highlight a ‘childhood advantage’ in memory consolidation (Ashtamker and Karni, 2013) and could suggest that the neural correlates of motor consolidation of a new motor routine into long-term memory are qualitatively different before and after the pubertal period.

Few studies have investigated the procedural learning and consolidation of a new motor routine in the context of neurodevelopmental disorders. With regard to learning itself, serial reaction time tasks (SRTT, Nissen & Bullemer, 1987) are particularly interesting to study a competition between a newly acquired sequence and a new one (proactive competition) because the SRTT paradigm requires to repeat a to-be-learnt sequence of finger tapping and to introduce suddenly a new but similar sequence. Typically, the performance (speed and accuracy) increases as the to-be-learnt sequence is repeated and decreases as the new sequence is introduced, hence revealing a competition between the new and the to-be-learnt sequence, which is the marker of implicit learning of the to-be-learnt sequence.

Two studies using the SRTT failed to find behavioural differences between typically developed (TD) and DCD children (Wilson, Maruff, & Lum, 2003; Lejeune, Catale, Willems, & Meulemans, 2013). Although the performance of the DCD group is globally lower than that of the TD group, both groups of children improved with practice of the repeated sequence and both groups presented a decrease in performance with the introduction of a new competitive motor sequence. In contrast, the study of Gheysen, Van Waelvelde, & Fias (2011) found an impairment in procedural learning in DCD children, with less decrease in performance as the introduction of the new sequence after practice of the to-be-learnt sequence. The apparent discrepancy between the results could refer to a difference in methodological factors such as taskcomplexity^1^ (Lejeune et al., 2013). Hence, a procedural learning deficit, which highlights a default in the competition between the learnt and a new sequence because the newly learnt motor routine is not strong enough to compete with a new subsequent motor routine, is found in the DCD group only when the task is complex enough.

With regard to consolidation, two studies used perceptual-motor tasks to investigate how a newly learnt motor routine evolves after practice. The study of Lejeune, Wansard, Geurten, & Meulemans (2015) found that, although the performance of children with DCD remained lower than that of TD children throughout the procedural learning of an inverted mouse task, the offline improvement of performance during the consolidation stage was similar in the two groups. In contrast, using a fine motor tracing task, the study of Zwicker, Missiuna, Harris, & Boyd (2011) revealed a larger difference in accuracy between TD and DCD children at retention (day 5) than at early practice (day 1), hence suggesting a default in the consolidation process in DCD children. The apparent discrepancy between the results suggests that the possible impairment in memory consolidation in DCD children may be task-dependent. Further investigations are required to understand which conditions influence the competition between the learnt and the new sequences in the DCD and TD group.

At the neural level, the model of Doyon and Benali (2005) predicts a specialisation of cortico-subcortical loops involved in learning as practice proceeds. The loops are recruited as a function of the type of task. Motor sequence learning, which corresponds to the acquisition of repetitive movements with practice (such as SRTT), requires the cortico-striato-cortical loop, whereas perceptual-motor adaptation, which corresponds to the increased capacity to compensate for environmental changes with practice, recruits the cortico-cerebello-cortical loop. To our knowledge, no study has yet compared the two types of learning and their neural correlates in DCD children (see Nicolson & Fawcett, 2007, for predictions). However, similarities between the difficulties found in perceptual-motor adaptation tasks in DCD children and patients with cerebellar lesions suggest a cerebellar dysfunction in DCD (Brookes, Nicolson, & Fawcett, 2007; Cantin, Polatajko, Thach, & Jaglal, 2007; Kagerer, Bo, Contreras-Vidal, & Clark, 2004). This assumption was comforted by the study of Zwicker et al. (2011), which revealed that compared to TD children, DCD children demonstrated an under-activation in the cerebellum on a retention test following practice of a fine motor task. To our knowledge, no study has yet reported direct evidence of an alteration of cortico-striatal circuitry in DCD (for a review of neural correlates of DCD, see Zwicker, Missiuna, Harris, & Boyd, 2009; Bo & Lee, 2013). Further studies are needed to investigate the possible alterations in motor learning-related circuits (cortico-striatal and cortico-cerebellar loops) in children with DCD to understand the possible impairments in learning and consolidation, which would suggest troubles in competition between old and new motor routines.

## Conclusion

Motor behaviour is a complex phenomenon encompassing different types of processes. It requires both flexibility and stability. Flexibility is required because a skilled behaviour adjusts to a changing context. Thus, generating new plans for action allows for adaptive performance in novel circumstances. Stability is required because similar contexts and tasks benefit from similar solutions (Clearfield, Diedrich, Smith, & Thelen, 2006). Big numbers of motor actions that would be appropriate for one situation might cause chaotic behaviour. The system must be structured so that it could achieve its goals by using only a subset of its behavioural repertoire at any instant (Rothkopf & Ballard, 2010). Motor memory could be viewed as a solution that may solve this problem of multiple candidate motor actions. Motor memory, viewed as an inherent property of the motor system, may stabilise or destabilise motor behaviour as a function of constraints.

The developmental trajectory of numerous motor skills is characterised by the transition between high variability and low variability. For instance, Kahrs, Jung, and Lockman (2012) studied developmental changes in movement parameters of infant banging. The developmental pattern they observed between 7 and 14 months shows clear changes in the spatial features, especially a decrease in the number of sideways, and forward movements of the hand. How does the behavioural stability emerge from the behavioural exuberance? According to Deutsch and Newell (2005), the reduction in children's performance variability with advancing age is primarily due to the evolving constraints of development and experience-driven changes in the adaptive structure of their sensory-motor input. These authors indeed consider the age-related reductions in the amount of variability during the performance of perceptual-motor tasks as a reflection of the changing constraints of development and enhanced ability through practice and experience to use available feedback information more effectively. However, motor memory could also play a key role. Van Swieten et al. (2010) consider that motor planning works as a blind watchmaker with actions reflecting a previous history of motor evolution where useful actions have survived and less useful ones have perished.

The studies discussed in the present article investigated the nature of the relationship between motor routines, action selection, and procedural learning in children with typical and atypical development. Such studies suggest that (1) the desirable equilibrium between stability and flexibility maintained by a mature behaviour can be achieved by a system that forms motor routines and (2) the level of equilibrium between persistence and flexibility of motor routines is an index of the maturity of the motor system.

## Footnotes

1 Wilson and Lejeune used unimanual SRTT tasks in which children had to tap with the four fingers of their dominant hand, while Gheysen required to tap with the index and middle fingers of both hands. Previous studies suggested that children with DCD present difficulties in improving performance on complex motor tasks (Marchiori, Wall, & Bedingfield, 1987) whereas they present similar improvements than TD children on simpler tasks (Revie & Larkin, 1993). The bimanual version of the SRTT task required in the study of Gheysen et al. (2011) could be considered as more difficult than the unimanual version used by Wilson and Lejeune because it involves interhemispheric transfer of information, which is known to be impaired in DCD children (DeGuise & Lassonde, 2001; Sigmundson, Ingvalsen, & Whiting, 1997; Sigmunddon & Whiting, 2002; Tallet, Albaret, & Barral, 2013).
